# Seasonal and Interseasonal Dynamics of Bluetongue Virus Infection of Dairy Cattle and *Culicoides sonorensis* Midges in Northern California – Implications for Virus Overwintering in Temperate Zones

**DOI:** 10.1371/journal.pone.0106975

**Published:** 2014-09-12

**Authors:** Christie E. Mayo, Bradley A. Mullens, William K. Reisen, Cameron J. Osborne, E. Paul J. Gibbs, Ian A. Gardner, N. James MacLachlan

**Affiliations:** 1 Department of Pathology, Microbiology and Immunology, School of Veterinary Medicine, University of California Davis, Davis, California, United States of America; 2 Department of Entomology, University of California Riverside, Riverside, California, United States of America; 3 Center for Vectorborne Diseases, University of California Davis, Davis, California, United States of America; 4 College of Veterinary Medicine, University of Florida, Gainesville, Florida, United States of America; 5 Department of Health Management, Atlantic Veterinary College, Charlottetown, Prince Edward Island, Canada; Institut Pasteur, France

## Abstract

Bluetongue virus (BTV) is the cause of an economically important arboviral disease of domestic and wild ruminants. The occurrence of BTV infection of livestock is distinctly seasonal in temperate regions of the world, thus we determined the dynamics of BTV infection (using BTV-specific real time reverse transcriptase polymerase chain reaction) among sentinel cattle and vector *Culicoides sonorensis* (*C. sonorensis*) midges on a dairy farm in northern California throughout both the seasonal and interseasonal (overwintering) periods of BTV activity from August 2012 until March 2014. The data confirmed widespread infection of both sentinel cattle and vector midges during the August – November period of seasonal BTV transmission, however BTV infection of parous female midges captured in traps set during daylight hours also was detected in February of both 2013 and 2014, during the interseasonal period. The finding of BTV-infected vector midges during mid-winter suggests that BTV may overwinter in northern California by infection of long-lived female *C. sonorensis* midges that were infected during the prior seasonal period of virus transmission, and reemerged sporadically during the overwintering period; however the data do not definitively preclude other potential mechanisms of BTV overwintering that are also discussed.

## Introduction

Arthropods and the viruses (arboviruses) they transmit are highly sensitive to seasonal changes in temperature and other climatic variables that can alter insect physiology and behavior as well as the kinetics of viral replication [1], [2], [3]. Bluetongue virus [BTV] is the cause of bluetongue, a globally emerging arboviral disease of domestic and wild ruminants that is transmitted by certain species of biting *Culicoides* midges [3], [4], [5]. These insects occur throughout most tropical and temperate regions of the world. It has been proposed that the recent emergence of BTV in Europe is linked to climate change, likely through the effects of temperature on the biology of the midges that serve as biological vectors of BTV and the rate of BTV replication within its insect host [3], [6], [7]. Bluetongue virus infection of animals at temperate latitudes is distinctly seasonal (typically July – November in the Northern Hemisphere) but it remains uncertain how BTV persists during winter months when cold temperatures restrict vector activity and reproduction [8], [9]. The mechanism of this so-called “overwintering” of BTV in endemic temperate regions has vexed scientists since the original published descriptions of BT in South Africa over a century ago [10]. Even the term overwintering is somewhat misleading because the interseasonal period of transmission cessation typically is greater than 6 months [8], [10], [11], [12].

Bluetongue was first identified beyond Africa and the Mediterranean Basin over 60 years ago when the disease was described in the USA [13], [14]. Ongoing surveillance since that time, including our studies, has confirmed that BTV infection of ruminant livestock in California is endemic but highly seasonal (late July – November), thus the interseasonal period (November – July) when BTV is apparently “inactive” lasts approximately 8 months [9], [15], [16], [17]. In the current study we undertook intensive surveillance of sentinel cattle and vector midges during both the seasonal and interseasonal periods of BTV activity at a dairy farm in Northern California (latitude 39.590° N). The goal of this surveillance effort was to characterize the interseasonal maintenance and dynamics of BTV activity throughout the entire calendar year, both in vector insects and sentinel cattle.

## Methods

### Detection of BTV infection of *Culicoides sonorensis (C. sonorensis)* midges

A longitudinal study of BTV infection of *C. sonorensis* midges and sentinel cattle on a dairy farm in northern California began August, 2012, as previously described [18]. Briefly, traps were placed at 0.25 km intervals along 4 transects, each directed at 90° quadrants from the outside perimeter of the waste-water lagoon. All but 4 traps were placed within open pasture or near forested areas with native trees. The remaining 4 traps set along the Eastern transect were adjacent to concrete free stalls (37×19 m; 1 roof; 4 open side structure). These free stalls are where most of the cattle are housed thereby allowing the potential opportunity for indoor feeding (endophagic) activity, an observation that has been documented among different *Culicoides* species within different regions of the world [19], [20], [21]

For harvesting of vector midges, we operated CDC style traps without light and baited with dry-ice [22] from dusk to dawn during both the seasonal (July – November) and interseasonal (December-June) periods and throughout the day only during the interseasonal period. Collections of *C. sonorensis* midges were made every other week for 52 weeks until August, 2013, as previously described [18]. Collection of adult midges was repeated during a portion of the interseasonal period of 2014 (January-March) on the transect where 32 parous females had been collected in 3 different traps during Feb 2013, and on another transect where no interseasonal activity of midges was detected during 2013. Trapping was performed weekly in 2014 with the addition of 3 tent - type emergence traps set and rotated weekly along the edge of the waste-water lagoon, a known larval habitat [9]. The emergence traps were constructed of no-seeum netting (Bioquip Products) securely fastened over polyvinyl chloride (PVC) pipe. Each trap was 2.0 m in length, 1 m in height and 2.0 m wide, centered at the mud-water interface of the gently sloping embankment so that 1 m was secured by stakes in the waste-water pond and 1 m was secured on the dry soil. A fitted 1-liter clear polystyrene jar was secured tightly to a 10 cm opening at the top of the trap and a conical piece of clear plastic was formed into a cone to impede exit of *C. sonorensis* collected in the container.

*Culicoides* midges were the only species identified in this trapping effort and were distinguished from other insects on the basis of their morphological characteristics and wing patterns using a stereomicroscope [23]. The *Culicoides variipennis (C. variipennis)* complex is separated into 3 discrete species: *C. sonorensis*, which is the primary vector of BTV in the western regions of North America and often associated with waste-water habitats; *C. occidentalis*, also a western North American species and typically localized to very saline or alkaline habitats; and, *C. variipennis*, which is present in eastern North America [24]. Females of *C. sonorensis* cannot be morphologically distinguished from *C. occidentalis*; therefore, the identity of female *C. sonorensis* was further supported by microscopic examination of a subset of slide-mounted male midges collected in the same traps from a random selection of traps in the transect.

*C. sonorensis* midges were sorted by sex and parity and then pooled into groups of 20 midges from the same collection date and trap number. All parous females and a subset (10% of each trap night) of pooled male and nulliparous midges were homogenized in lysis binding buffer (AM8500, Ambion/Life Technologies, Grand Island, NY) with a lysing matrix of stainless steel beads processed in a homogenizer (Bullet Blender STORM, Next Advance Inc., Averill Park, NY). Viral RNA was extracted from the homogenate using the viral RNA isolation kit (AM1836, Ambion) according to the manufacturer's recommendations. The presence of BTV in the homogenated midge pools was determined by quantitative reverse transcriptase polymerase chain reaction (qRT-PCR) amplification and detection of the BTV S10 gene, as previously described [9]. Lysates of pooled *C. sonorensis* midges collected during the interseasonal period were run in duplicate and the assays repeated to confirm the findings.

### Detection of BTV infection of sentinel cattle

Cattle resident on the dairy farm were used as sentinels to detect circulation of BTV from August 2012 until August 2013. Individual groups of cattle were enrolled as two separate cohorts at two different times during the study. Cattle in the first cohort (cohort 1) were dairy heifers (6 months of age, female) and free of BTV infection as determined by virus-specific qRT-PCR and competitive ELISA (cELISA) assays. A total of 35 heifers were enrolled in cohort 1 in July, 2012, during the seasonal period of BTV transmission. Two of the heifers were lost from the study during the year due to diarrhea and trauma events, leaving 33 cattle from which blood/sera were collected bi-weekly until August, 2013, during the following BTV transmission season. Twenty-nine calves were enrolled in the second cohort (cohort 2). The animals in cohort 2 were all female calves born on the farm in January, 2013, which was during the interseasonal period of virus activity. Blood and sera were collected within one day of birth and then bi-weekly until August, 2013. The calves enrolled during the interseasonal period were all uninfected as determined by BTV-specific qRT-PCR assay but seropositive to BTV by cELISA, which was attributed to the consumption of colostral antibodies [18]. These calves were all seronegative to BTV by May of 2013, prior to the anticipated BTV transmission season. Calves in cohort 2 were housed together for the first 4 months of life, whereas the sentinel cattle in cohort 1 were dispersed throughout the farm at selected locations that were based on previously published data that showed variation in BTV infection status of cattle with proximity to anthropogenic variables such as dairy waste water lagoons, a known breeding habitat of *C. sonorensis*, or free stalls and barns that potentially could support adult midge populations during the winter months [15], [19]. Additional sentinel cattle were enrolled to further investigate potential BTV infection of cattle on the farm during the interseasonal period of 2014, specifically: 30, 8 - month old heifers were evaluated once (cross section) by qRT-PCR in February, 2014. An additional cohort of 15 calves were enrolled during January 2014 and monitored until March, 2014.

Whole blood and serum collected from animals in each cohort were stored (whole blood -80°C, serum −20°C) until the end of the study year when samples were tested for BTV-specific antibodies using a commercial cELISA (VMRD, Pullman, WA). Whole blood samples from animals that tested positive by cELISA were then screened for the presence of viral RNA by BTV-specific qRT-PCR so that the approximate date (by week) of BTV infection could be estimated based on the date of the first blood collection in which viral RNA was detected, as previously described [9].

### Data analysis

Midge infection rates per 1,000 parous female *C. sonorensis* and 95% confidence intervals were calculated using the bias-corrected maximum likelihood estimates (MLEs) by using the Excel Add-In PooledInfRate, version 3.0 [25]. The mean Ct value was calculated from the population of parous female *C. sonorensis* or sentinel cattle that had Ct values <40, which is the maximum number of cycles within our qRT-PCR assay.

## Results

**Figure 1:**
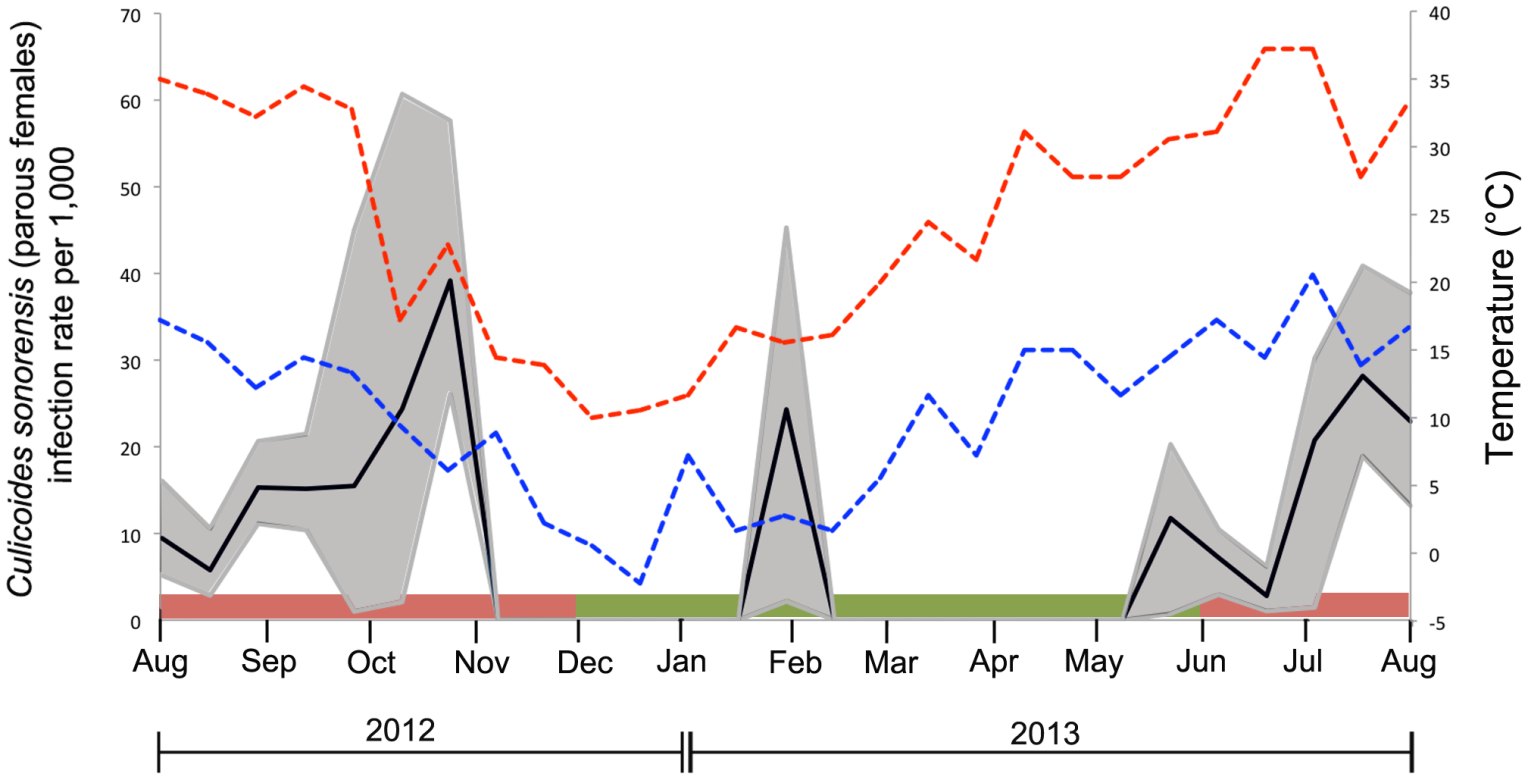
Seasonal patterns of BTV infection among parous female *C. sonorensis* midges and sentinel cattle collected on a dairy farm during the period of August 2012-August, 2013. The point estimate (solid line) of infection rate per 1,000 tested *Culicoides*; 95% confidence intervals (shaded region); maximum temperature (°C - red dash line); minimum temperature (°C – blue dash line). Shaded regions at the base of the graph are representative of historically described seasonal (red shading) and interseasonal (green shading) periods of infection.

**Figure 2:**
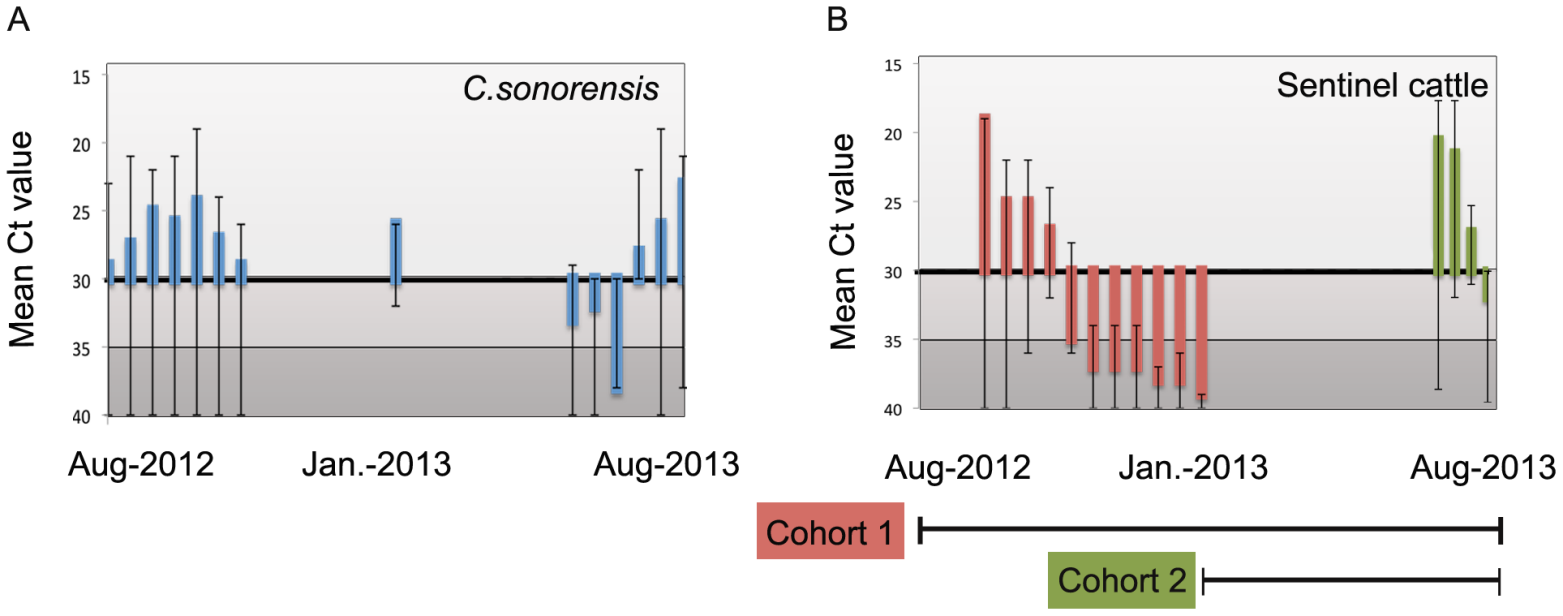
Seasonal detection of BTV RNA among parous female *C. sonorensis* midges; blue histogram represent mean cycle threshold (Ct) values obtained from qRT-PCR assays. Panel A. Seasonal detection of BTV RNA among sentinel cattle enrolled in two cohorts; Panel B. Cattle in cohort 1 (red histogram) were enrolled during the seasonal period of infection and monitored throughout the year (July, 2012-August, 2013) while cattle in cohort 2 (green histogram) were enrolled during the interseasonal period and monitored throughout the season (January 2013-August, 2013). In each figure, the scale on the y-axis is inverted where 15 represents the upper limit and 40 the lower limit and error bars are indicative of the maximum and minimum Ct values for each date. The solid line indicates the threshold for interpretation of a positive result (light grey; Ct range 15–30). Below the black line, Ct results are interpreted as indeterminant (medium grey; Ct range 30–35) and negative (dark grey; Ct range 36–40).

The duration (July – November) and peak occurrence (late September) of seasonal BTV infection among *C. sonorensis* midges were similar to those described in our previous studies [9], [17], [26] (Figures 1&2). Similarly, the duration of seasonal BTV infection among sentinel cattle in cohort 1 (September-February) as determined by qRT-PCR was similar to that described in our previous studies (Figure 2). Within cohort 1, 3 cows were infected in September, 2012, 21 cows were infected in October, 2012 and 9 were infected in November, 2012. It is to be stressed that the Ct values in the cattle in cohort 1 were low (strongly positive) during the seasonal period of virus activity and became increasingly elevated (weaker) during the interseasonal period (after November, 2012) when the weakly positive results likely represented the prolonged presence of non-infectious viral RNA [27], [28]. Consistent with the literature, infectious BTV is usually associated with Ct values of <30 (unpublished data) and Ct values of positive cattle in cohort 1 during the seasonal period of BTV transmission typically ranged from 19–28, whereas during the interseasonal period Ct values among cattle in this cohort ranged from 30–38 [29], [30]. Only one of the 29 calves enrolled during January 2013 (cohort 2) became qRT-PCR positive (Figure 2). This calf was strongly positive (Ct 20) in June and Ct values increased by August, 2013 with subsequent seroconversion, indicating an active infection occurred in this calf beginning in June of 2013. Of the sentinel cattle that were enrolled during the interseasonal period of 2014, only 4/30 cattle had very weak Ct values (37–39), All 15 calves enrolled during 2014 were seropositive (colostral antibodies) but remained negative via qRT-PCR. Together, evaluation of the various sentinel animals confirmed the lack of virus transmission to sentinel cattle during the interseasonal periods of both 2013 and 2014.

Adult *C. sonorensis* were collected during the daytime on three separate dates during the interseasonal period of both 2013 and 2014: 1. 18 Feb 2013 (32 parous females); 2. 21 Feb 2014 (3 males, 3nulliparous females, 2 parous females); 3. 21 Mar 2014 (2 males, 17 parous females). Of the parous females collected, two pools were positive by qRT-PCR assay (Ct values of 26 and 27 respectively) and one pool had a high Ct value (38) indicating a very weak or negative result. *Culicoides* collected from the emergence traps included 8 males, 3 nulliparous females on 14 March 2014 and 5 males, 13 nulliparous females on 21 Mar 2014. All midge pools collected via the emergence traps were negative for BTV RNA via qRT-PCR assay. Furthermore, evaluation by qRT-PCR of nulliparous female and male midges collected throughout the year showed no evidence of BTV viral RNA.

## Discussion

The objective of this study was to describe the interseasonal maintenance and seasonal/interseasonal dynamics of virus infection of vector midges and cattle resident on a dairy farm in Northern California with endemic but highly seasonal BTV infection. For the first time, this study documented the presence of BTV RNA in pools of parous female *C. sonorensis* midges collected during the interseasonal period in 2013 and again in 2014, which potentially explained the mechanism for overwintering of BTV on this farm. The detection of BTV in pools of parous female midges collected host-seeking during the day in the interseasonal periods of both years most likely reflects one of the following mechanisms: 1) long-lived female midges that were infected during the prior seasonal period of virus transmission, which were less active in the fall and re-emerged in midwinter during a transient period of higher temperature; or 2) uninfected female midges that emerged during February-March from cohorts oviposited in the fall to feed on a BTV-infected animal, although animal surveillance at the time identified no active virus circulation among an extensive cohort(s) of sentinel cattle. Other less likely but potential explanations for the presence of virus-infected parous female midges in mid-winter include: 3) uninfected larval stages that remained viable throughout the winter to emerge as host-seeking females in midwinter that then fed on viremic hosts and then oviposited, or 4) vertical transmission of BTV via immature stages to produce infected host-seeking female midges in the winter. Weighing the evidence, we consider that mechanism 1 represents the most probable explanation.

This finding is globally relevant to temperate areas and to future incursive zones of BTV activity facilitated by climate change. Previously Nevill (1971) proposed potential methods for the interseasonal maintenance of BTV on the high veldt of South Africa, including: 1. transovarial transmission of BTV in vector insects; 2. a complicated overwintering cycle involving an unidentified intermediate host; 3. prolonged infection of ruminant livestock; 4. prolonged survival of adult *Culicoides* insects; 5. an ongoing and slow/low-level cycle of infection between cattle and *Culicoides* midges. Comments with regards to each of these potential explanations and our observations include:

1. Transovarial transmission of the virus from infected midge females to their F1 progeny. Although this phenomenon has been shown to occur at various rates in some arboviruses transmitted by mosquitoes and ticks, it has not been proven definitively for BTV with any *Culicoides* species [31], [32], [33]. Similarly, we did not identify BTV infection of either male or nulliparous female *C. sonorensis* collected during the interseasonal period although sample sizes were small.
2. A complicated overwintering cycle that involves some unidentified intermediate host such as reptiles or birds. This possibility seems unlikely in California because *C. sonorensis* appears to feed principally on ruminants. Analysis of blood-fed *C. sonorensis* midges collected during this project showed that these vectors fed only on cattle (*Bos taurus*) and Black - tailed deer (*Odocoileus hemionus columbianus*) (unpublished data).
3. Prolonged infection of ruminant livestock. Extensive studies in recent years confirm that the duration of BTV infection in livestock is considerably less than that of the approximately 6 - month interseasonal period. Although viral RNA persists for several months following BTV infection of ruminants, duration of the viremia that is infectious to vector midges is typically <60 days [27], [28], [34], [35], [36].
4. Prolonged survival of adult *Culicoides* insects infected following the period of active BTV transmission. Female midges are infected for life, but become infectious only after an extrinsic incubation period that is temperature dependent. The lifespan of BTV-infected vector midges in the field is unknown, but is less than 90 days under laboratory conditions [2], [37], [38]. However, much remains to be learned regarding temperatures at potential refugia where adult midges may rest in nature during the interseasonal period and their impact on midge survival.
5. An ongoing and slow/low-level cycle of infection and transmission between cattle or other ruminants and *Culicoides* midges throughout the interseasonal period.

During both 2013 and 2014, only parous female midges collected in CO_2_ traps used for our interseasonal collection during daylight hours were strongly positive for BTV viral RNA. BTV was not detected in either male or non-parous female midges and midges were not collected after sunset. The collection of substantial numbers of parous females only during daylight hours on a limited number of days during the interseasonal period contrasts with the “catch” obtained using conventional methods, i.e. light traps, that are typically set during dusk and run throughout the night when temperatures are lowest and few insects are active. BTV viral RNA was detected among sentinel cattle at the site through February, 2013, and again in February, 2014, but Ct values of RNA from positive cattle blood collected during winter months were high (Ct range 30–38) indicating few RNA copy numbers as compared to blood samples collected during the July – November period of active BTV transmission (range of Ct values, 19–28 in cattle blood, 25–29 in vector midges) in 2012. In contrast, the Ct values of the pools of parous midges collected in February, 2013, and March, 2014 were strongly positive (Ct <30). In our experience, infectious virus typically is associated with Ct values ≤30 in cattle blood.

A marked change in midge population age structure occurred with the transition from the late seasonal to interseasonal periods of BTV activity, with the majority of midges collected after late November, 2012 being parous females (70% of collections, as contrasted with 26–38% in prior months). Furthermore, concurrent sampling of dairy waste-water lagoons confirmed that midge larvae were not detected in edge mud after December, 2012 and that midge pupae (indicative of impending emergence of adult midges) were not found until July, 2013 [18]. In 2014, adult emergence was detected with emergence traps on two separate dates, but midges collected at those times tested negative for BTV viral RNA. This finding confirmed the low-level emergence of midges throughout the winter. However, we found no evidence of vertical transmission of BTV in these insects or in nulliparous females collected and tested during the active transmission period. Thus, vertical transmission would seem to play little or no role in the interseasonal maintenance or seasonal transmission of BTV infection on the farm.

In contrast, the presence of BTV in 2 pools of overwintering parous female *C. sonorensis* midges implicated adult parous midges in the interseasonal maintenance of BTV on the farm. The low Ct (26, 27) values of these midge pools is consistent with the presence of infectious BTV (Ct≤30), which indicated that BTV persisted in the vector at least through midwinter (although it does not necessarily imply fully disseminated infection) [39]. Gravid mosquitoes can survive >6 months within refugia in California and still transmit arboviruses [40], [41]. Adult *C. sonorensis*, once infected with BTV, apparently remain infected for life [1], [42]. Although the maximal lifespan of BTV-infected parous female midges in the field remains unknown, the resumption of gonotrophic activity of BTV-infected adult midges following the interseasonal period could re-establish the transmission cycle in endemic temperate areas. Alternatively, but less likely, the adult *C. sonorensis* midges could have been infected by feeding on a viremic (recently infected) animal during the winter, completed a gonotrophic cycle, and were then collected when attempting to re-feed.

In summary, our findings strongly suggest that BTV infected adult *C. sonorensis* midges contribute to the maintenance of BTV through the interseasonal “overwintering” period in temperate regions, most likely by their prolonged survival until the next seasonal transmission period.
